# Cognitive-Behavioral Social Skills Training: Outcome of a Randomized Controlled Trial for Youth at Risk of Psychosis

**DOI:** 10.1093/schizbullopen/sgad020

**Published:** 2023-08-02

**Authors:** Jean Addington, Lu Liu, Amy Braun, Kali Brummitt, Kristin S Cadenhead, Barbara A Cornblatt, Jason L Holden, Eric Granholm

**Affiliations:** Department of Psychiatry, Hotchkiss Brain Institute, University of Calgary, Calgary, Alberta, Canada; Department of Psychiatry, Hotchkiss Brain Institute, University of Calgary, Calgary, Alberta, Canada; Department of Psychiatry, Hotchkiss Brain Institute, University of Calgary, Calgary, Alberta, Canada; Department of Psychiatry, Hotchkiss Brain Institute, University of Calgary, Calgary, Alberta, Canada; Department of Psychiatry, University of California, San Diego, San Diego, CA, USA; Department of Psychiatry, Zucker Hillside Hospital, Long Island NY, USA; Department of Psychiatry, University of California, San Diego, San Diego, CA, USA; Department of Psychiatry, University of California, San Diego, San Diego, CA, USA; Veterans Affairs San Diego Healthcare System, San Diego, CA, USA

**Keywords:** psychosis, clinical high risk, social functioning, role functioning, CBSST

## Abstract

**Aim:**

Difficulties in social functioning have been observed in youth at clinical high-risk (CHR) of psychosis even in those who do not go on to develop a psychotic illness. Few treatment studies have attempted to improve social functioning in this population. The aim of this study was to conduct a randomized trial comparing the effects of Cognitive-Behavioral Social Skills Training (CBSST) with a supportive therapy (ST).

**Methods:**

Both CBSST and ST were weekly group therapies, delivered over 18 weeks. This was a 2-arm trial with single-blinded ratings and intention-to-treat analyses. Assessments occurred at baseline, end-of-treatment, and 12 months after the baseline assessment. The primary outcome was social and role functioning and defeatist performance attitudes were the secondary outcome. Attenuated positive and negative symptoms, anxiety, depression, self-efficacy, and beliefs about self and others were examined as exploratory outcomes.

**Results:**

There were no significant differences between the 2 groups at baseline or either of the 2 follow-ups. However, at follow-ups, in each group there were significant improvements in clinical symptoms. These could not be attributed to group treatment since there was no control or wait-list group.

**Conclusions:**

Since poor social functioning is one of the most observed difficulties in CHR individuals, and a decline in social functioning may be a significant predictor of later transition to psychosis, future work will be needed to find effective treatments for this decline in functioning for CHR youth.

## Introduction

An important direction in schizophrenia research has been the focus on youth at clinical high-risk (CHR) for psychosis, with a key goal being the prevention of transition to full-blown psychosis. CHR individuals present with a wide range of clinical problems, and only a small percentage develop a full-blown psychotic disorder.^1^ Many CHR individuals who do not go on to develop psychosis remain troubled by fluctuating symptoms and poor social and role functioning.^2^ These youth are characterized by long-standing cognitive, social, and role deficits that, if left untreated, could potentially lead to significant disability, regardless of the presence or severity of psychotic-like symptoms.^3^ Moreover, poor social functioning, particularly a decline in social functioning, increases the likelihood of transitioning to psychosis.^4^

In schizophrenia research, it has been noted that pharmacologic treatments have had minimal impact on functional impairment, although psychosocial treatments have been moderately effective, with social and role functioning considered important treatment outcomes.^5^ Typically, both transition to psychosis and reduced attenuated psychotic symptoms are primary outcomes in most CHR treatment trials; however, improving functioning should be considered equally important.^6^ A recent meta-analysis of randomized controlled trials (RCT) examining social functioning in CHR samples concluded that none of the available treatments significantly improved social functioning in those at CHR.^7^ Some trials did report improvement in social functioning in both the control and experimental groups^8,9^ and the multi-site NEUROPRO study reported improvement for those receiving long-chain omega-3 polyunsaturated fatty acids (n-3 PUFAs) on the Social and Occupational Functioning Assessment Scale (SOFAS) but not Global functioning: Social (GF:S) compared to the placebo control group.^10^ However, in this review it was apparent that very few if any treatment trials for CHR had social and/or role functioning as their primary outcomes. More importantly, the interventions were not chosen or designed to specifically target functional outcomes.^11^

Since the review of Devoe et al,^7^ there have been additional trials, aiming to improve social functioning, published. In a small pilot trial (*n* = 38) comparing an integrated social and cognitive remediation therapy, Cognition for Learning and for Understanding Everyday Social Situations (CLUES), with an active control intervention it was reported that at the 3-month follow-up of 23 participants, there was a significant improvement in social functioning for those in CLUES.^12^ In contrast, Glenthoj et al^13^ found no significant improvement in social functioning after 20 weeks of cognitive remediation therapy compared to treatment as usual. In the NEUROPRO study cited above, recent results demonstrated that those receiving omega-3 fatty acids had improved functioning at both 6 and 12 months, but again, only on the SOFAS.^14^ Finally, Myin-Germeys et al^15^ reported that when acceptance and commitment therapy was added to treatment as usual, there was a significant improvement in functioning assessed with the SOFAS and Social Functioning Scale at follow-up. Again, except for the pilot feasibility study by Friedman-Yakoobian et al^12^ none of these trials had social functioning as their primary outcome. Thus, there is a need for clinical trials to specifically target social and role functioning in CHR individuals.

In considering potential interventions to address social functioning, one to be considered would be Cognitive-Behavioral Social Skills Training (CBSST).^16^ CBSST is a group intervention, which combines elements of cognitive-behavior therapy (CBT) and social skills training (SST), both evidenced-based interventions for schizophrenia.^17^ By adding CBT to SST to target functioning outcomes, SST can be used to train new social skills, and thoughts that might interfere with functioning in the real world (eg, low self-efficacy, defeatist performance attitudes) can be addressed using CBT. Defeatist beliefs have been linked to amotivation and poor functioning in schizophrenia^18^ and reduction in severity of defeatist attitudes is associated with improvements in these outcomes in CBSST.^16^ There are several successful trials of CBSST in individuals with schizophrenia, including evidence of improved social and role functioning and its maintenance at 3-month follow-up after CBSST for a younger first-episode psychosis population.^19^ Based on the several studies demonstrating that individuals at CHR have impaired social and role functioning,^20–22^ an adapted version of CBSST, more appropriate for the ages and the severity of illness of typical CHR individuals, may improve social and role functioning for these young people.^23^

Thus, the trial described in this article is the Recovery through Group (Regroup) project, which is an effort to determine whether a group approach would help recovery. Our goal was to test the efficacy of our adapted CBSST psychosocial intervention for social and role-functioning difficulties in CHR youth. This clinical trial compares CBSST to a group supportive therapy (ST) treatment. Two previous publications describe in detail the methods of Regroup^24^ and the modifications for CHR youth to the published CBSST manual.^23^ Our primary aim was to examine whether CBSST improves social and role functioning and defeatist beliefs when compared to ST in youth at CHR and a secondary aim was to determine if there were any changes in other clinical symptoms. Our primary outcome was social and role functioning, the secondary outcome was defeatist beliefs and exploratory outcomes included attenuated psychotic symptoms, negative symptoms, depression, anxiety, and self-perceptions.

## Methods

### Design

Regroup was a NIMH-funded, 5-year, 3-site study conducted at the University of Calgary, Canada, the University of California, San Diego (UCSD), USA, and Zucker Hillside Hospital in New York, USA. The trial was registered at ClinicalTrials.gov, registration number NCT02234258. The methods of this trial have been described elsewhere in more detail.^24^ It was a single-blind RCT of CBSST vs ST with an 18-week group treatment phase and an end-of-treatment and 12-month post-baseline follow-ups. After baseline 203 participants were randomized using concealed stratified randomization with minimization.^25^ Participants were stratified by sex and whether they were receiving an antipsychotic. Clinical ralters were blind to the treatment group. CBSST and ST groups were delivered by the same therapists for the same number of sessions and length of time. Both therapies were manualized.

### Participants

Recruitment of participants was sought from health care providers, educators, or social service agencies or they were self-referred in response to educational efforts in the community. Potential participants were first screened by phone and if they appeared to meet inclusion criteria attended an in-person eligibility and consent evaluation.

All participants either currently or had in the past 2 years met the Criteria of Psychosis-Risk Syndromes (COPS) which is based on the Structured Interview for Psychosis-Risk Syndromes (SIPS).^26^ After a comprehensive assessment that included administering the Structure Clinical Interview for DSM-5 (SCID) and the SIPS version 5.6, vignettes were written and presented on a call for a consensus decision on the CHR diagnosis and symptom ratings. These weekly consensus calls, chaired by J. Addington, were attended by clinical raters from all 3 sites.

For inclusion criteria CHR participants were between 12 and 30 years old and met diagnostic criteria for a psychosis-risk syndrome as per the COPS criteria^26^ either currently (COPS progression) or at some point in the past 2 years and continued to have attenuated psychotic symptoms (COPS persistence). Participants rated 7 or less (see below under measures) on either of the Global Functioning Scales: Social and Role (GF:S, GF:R).^27,28^ Exclusion criteria included (1) meeting criteria for current or lifetime psychotic disorder, including affective psychoses, (2) IQ less than 70, (3) history of a central nervous system disorder, or (4) diagnostic psychosis-risk symptoms were clearly caused by another disorder. Other non-psychotic DSM-5 disorders were not exclusionary (eg, substance-related disorders, major depression, anxiety disorders, personality disorders), provided the disorder did not account for the individual’s psychosis-risk symptoms. The use of antipsychotics was not an exclusion, provided there was clear evidence that psychosis risk, but not psychotic symptoms, were present when the medication was started. Participants could not have been involved in a cognitive-behavior therapy in the last 12 months.

It should be noted that, at baseline, 4 participants (2 in each treatment arm) who had met COPS criteria for attenuated psychotic symptom syndrome in the past year did not meet current COPS criteria since in the past month they only rated 2 (mild) on their SOPS positive symptoms. For a SOPS symptom to meet attenuated symptom criteria, it has to be rated 3–5. They did have social functioning ratings of 6 or lower and were therefore included.

### Measures

The Structured Clinical Interview for DSM-5 (SCID-5)^29^ was used to determine the presence of Axis I disorders and the SIPS^26^ to determine if the criteria for a psychosis-risk syndrome were met.

Functioning (primary outcome) was assessed with the Global Functioning Scales: Social and Role (GF:S, GF:R)^27,28^ which provides a single score for social and a single score for role functioning that ranges from 1 to 10 (eg, 1 = extreme social isolation or extreme role dysfunction, 5 = serious impairment, 6 = moderate impairment and 7 = mild problems in social or role function). The GF:S and GF:R were specifically designed for CHR youth to measure changes in functioning across time.^27,28^ A decline in social functioning in the year before entering the study was included as a leading predictor of psychosis in the NAPLS Psychosis-Risk Calculator.^30,31^ Defeatist beliefs (secondary outcome) were assessed with the Defeatist Performance Attitude Scale.^32^

Exploratory measures included the Scale of Psychosis-Risk Symptoms (SOPS),^26^ to assess severity and onset of psychosis-risk symptoms, the Calgary Depression Scale for Schizophrenia^33,34^ for depression, and the Social Interaction Anxiety Scale^35^ and the Social Anxiety Scale^36^ to assess anxiety.

For other beliefs and attitudes, the following measures were used: The Brief Core Schema Scales^37,38^ to assess negative and positive beliefs about self and others, the Asocial Beliefs Scale^18^ to assess social disinterest in interacting with others, and the Social Self-Efficacy scale (SSSE)^39^ to assess self-efficacy expectations.

At baseline 2 subscales of the Wechsler Abbreviated Scale of Intelligence-II (WASI-II)^40^ were used to estimate current IQ: Vocabulary and Matrix Reasoning.

### Interventions

Granholm and colleagues developed, manualized and in a range of studies tested the efficacy of CBSST; an intervention that integrates CBT and SST which has been well described in a practical treatment manual.^16^ CBSST is an 18-week group comprised of 3 modules; (1) Cognitive Skills, (2) Social Skills, and (3) Problem-Solving Skills. Each module includes 6 weekly group sessions and allows rolling entry at the beginning of each module. The CBSST manual^16^ was adapted to make the content more appropriate for the age range and symptom severity of CHR youth. Key changes included a focus on normalization and DE stigmatization of attenuated psychotic symptoms, as well as examples of thought challenging, role-plays, and problem-solving that were more appropriate for this younger CHR population. A detailed description of the key changes has been reported in Kelsven et al^23^ with a summary presented in supplementary material.

An 18-week ST group that also allowed rolling entry every 6 weeks served as the control treatment, to match CBSST for the nonspecific effects of therapist and peer group contact and interest, social interaction, and support. There were brief guidelines as to what therapists could and could not do. Typically, each session opened with the therapists asking how the previous week had been. If there had been crises these were addressed, and advice was given from therapists and members to help with any immediate or ongoing problems. No active CBT or SST techniques were taught or used and educational material about CHR for psychosis was offered. The therapists focused on listening, reflecting, and empathizing, and demonstrating uncritical acceptance and genuineness. Social interaction among participants was encouraged. Participants in the ST group were not given any homework.

All therapy sessions were delivered by master or doctoral-level therapists under the supervision of Dr. Eric Granholm. There were co-facilitators for each group. Two days of training including standardized video demonstrations, and role-play practice with coaching was conducted at UCSD by Dr. Granholm prior to the start of the trial, followed by weekly videoconference supervision with therapists from all 3 sites.

### Treatment Fidelity

All sessions were audio-recorded. These recordings plus any fidelity ratings of sessions were used in the weekly supervision sessions to introduce technical modifications between sessions and provide therapists feedback to improve fidelity. Eighty-seven randomly selected audio recordings of CBSST sessions (stratified by site and module) were rated for fidelity using the Cognitive Therapy Rating Scale for Psychosis (CTS-Spy)^41^ and the Social Skills Group Observation Checklist (SSGOC).^42^ Six items related to role-play practice were rated from the SSGOC (established a rationale, discussed and modeled steps, engaged client in a role play, provided positive feedback, provided suggestions for improvement, and reinforced small steps in repeated role plays; all rated 0, absent, or 1, present), because the remaining items overlap with nonspecific therapist items (eg, understanding, interpersonal effectiveness) and session-structure items (eg, agenda setting, at home practice) which were also rated on the CTS-Spy.

### Procedures

Raters were experienced research clinicians who demonstrated excellent reliability when compared to “gold standard” ratings on the SOPS (ICC = 0.91), GF:S (ICC = 0.89) and GF:R (ICC = 0.89). This study was approved by the Institutional Review Boards of all 3 sites. Written informed consent, including parental consent, was obtained from all adult participants and parents/guardians of minors. A Data and Safety Monitoring Board (DSMB) were set up with NIMH-approved members. Calls with the DSMB and site PIs occurred every 4 months. All groups were in-person.

### Statistical Analysis

All descriptive comparisons used independent samples *t*-tests for continuous variables and chi-square tests for categorical variables. The power calculation recommended 62 completers per arm of the final follow-up of the primary and secondary outcomes. The power analysis is presented in supplementary material.

To accommodate missing data and account for intra-participant correlation over time, a generalized linear mixed model for repeated measures was used to examine changes over time (baseline, EOT, 12 months) for between and within group differences on the primary outcome social and role functioning, the secondary outcome defeatist beliefs and the exploratory clinical variables (positive, negative, depression, and anxiety symptoms) and measures of self-perception. Although transition to psychosis was not an outcome in this study survival probability over time was computed using the Kaplan–Meier estimate.

All statistical analyses were conducted using the IBM Statistical Package for the Social Sciences (SPSS) version 25 and Statistical Analysis Software (SAS) version 9.4.

## Results

### Participants

**Fig. 1.**
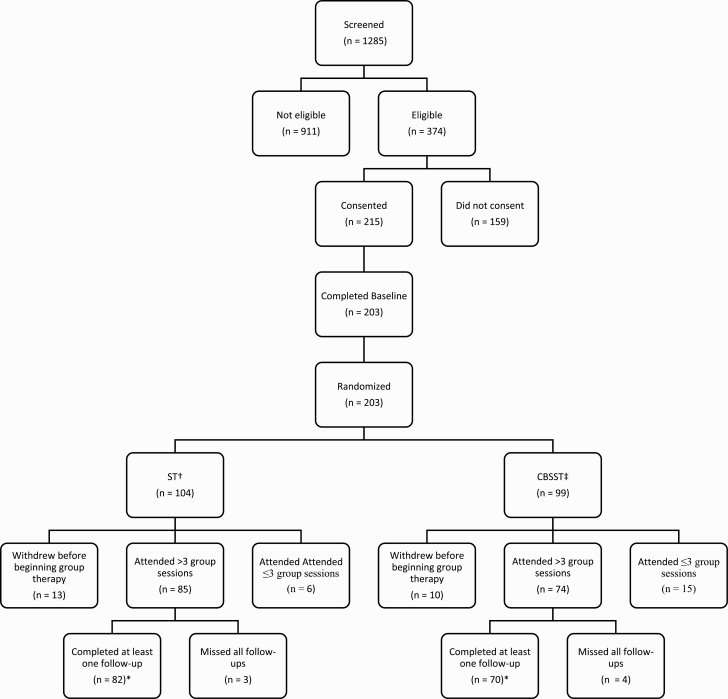
Consort diagram. † Supportive therapy ‡ Cognitive-behavioral social skills training *Final numbers used for analysis

Details of recruitment and follow-up are presented in the Consort Diagram in figure 1. Of the 215 participants who consented, 203 completed the baseline assessment and were subsequently randomized. Of the 203 randomized, 23 withdrew before beginning therapy. A further 21 attended 3 or less sessions (17 had no follow-up sessions, 4 attended only 1 or 2 sessions but did have one follow-up). Unfortunately, 7 participants did attend several group sessions (range 5-15) but were unavailable for any follow-up assessments. These 51 (25%) participants were not included in the final analyses (CBSST = 29, ST = 22). Thus, the final sample for analyses included 152 participants (CBSST = 70, ST = 82).

Those who were excluded (N = 51) were compared to those included (*N* = 152). The groups did not differ significantly on any demographics, social or role functioning, IQ, symptoms, or self-perception variables. Those who were excluded were compared to those included in separate analyses for CBSST and ST and again there were no differences (see supplementary tables 1–3).

Thirty-four participants were taking an antipsychotic at baseline with 28 and 29 respectively at the 2 follow-ups. Not all participants were on an antipsychotic for the duration of the study in that some began during treatment and others sometime prior to the 12-month follow-up. Only 10 participants were on an antipsychotic at baseline and in all subsequent follow-ups. Details of medications are presented in supplementary table 4.

There were no differences in any demographics between the treatment groups. See table 1.

**Table 1.** Demographic Comparisons at Baseline

Variable	CBSST *n* = 70	ST *n* = 82	Test Statistic	
	Mean (SD)	Mean (SD)	t	P-value
Age (years)	17.36 (4.01)	17.49 (4.12)	−0.20	.844
Years of education	10.30 (2.68)	10.45 (2.64)	−0.35	.727
IQ	103.00 (12.24)	104.00 (14.90)	−0.48	.631
	*Frequency (%)*	*Frequency (%)*	χ^*2*^	*P-value*
Sex				
Male	29 (41.4)	40 (48.8)	0.82	.364
Female	41 (58.6)	42 (51.2)		
Race				
Caucasian	41 (58.6)	51 (62.2)	0.22	.895
Black	8 (11.4)	9 (11.0)		
Other^a^	21 (30.0)	22 (26.8)		
Marital status				
Single/never married	67 (95.7)	79 (96.3)	0.71	.701
Other	3 (4.3)	3 (3.7)		
Living arrangement				
Living with family	63 (90.0)	71 (86.6)	1.66	.646
Living with spouse/partner	3 (4.3)	5 (6.1)		
Living on own	2 (2.9)	1 (1.2)		
Living with others^b^	2 (46.1)	5 (6.1)		
Education completed				
Grade school	48 (68.6)	53 (64.6)	2.33	.675
High school	19 (27.14)	22 (26.8)		
College	3 (4.3)	6 (7.3)		
Technical school	0 (0.0)	1 (1.2)		
Current employment				
Working full-time	2 (2.9)	4 (4.9)	3.54	.315
Working part-time	15 (21.4)	9 (11.0)		
Worked in past year	15 (21.4)	17 (20.7)		
Not worked in past year	38 (54.3)	52 (63.4)		

^*a*^ Includes First Nations, East Asian, Southeast Asian, South Asian, West/Central Asian, and Middle Eastern, Native Hawaiian or Pacific Islander, Interracial.

^*b*^ Includes living with friends (excluding spouse/partners), in a boarding/group home, or academic residence.

There were no differences in the mean number of group sessions attended by each group (mean [SD] for CBSST = 13.8 [3.5], for ST = 13.8[3.3]).

### Treatment Fidelity

CTS-Psy total fidelity ratings did not differ significantly across sites, *F*(2,84) = 0.29, *P* = .75 (*M ± SD*: Site 1 = 42.28 *±* 4.50, Site 2 = 40.30 *±* 6.34, Site 3 = 39.57 *±* 7.04). The CTS-Psy total score was significantly greater in CBSST (40.76 *±* 6.04) relative to the control treatment of ST (18.56 *±* 1.79), *t*(85) = 14.49, *P* < .001, and was above the cutoff for competent CBT for psychosis (>30) used in previous clinical trials,^41^ (ie, for CBSST, but not for ST). The mean rating on the 6 SSGOC role-play items was significantly greater for the CBSST Social Skills Module (4.13 *±* 1.87) than for ST (0.00 *±* 0.00), *t*(69) = 15.46, *P* < 001. Thus, high-fidelity CBT and SST interventions were delivered in CBSST with comparable high-fidelity across the 3 sites. More details on fidelity can be found in Addington et al.^24^ and Kelsven et al.^23^

### Outcome

Table 2 presents the differences in social and role functioning, defeatist beliefs, and the exploratory clinical variables (positive, negative, depression, and anxiety symptoms) and measures of self-perception within and between the groups. The 2 treatment groups did not differ significantly on any outcome measure at baseline, end of treatment nor at 12 months.

**Table 2.** Differences in Clinical Variables Within and Between Groups

Variables	CBSST (n = 70)			ST (n = 82)		
	Baseline (*n* = 70)	End of Treatment (*n* = 66)	12 months *(n* = 57)	Baseline *(n* = 82)	End of Treatment (*n* = 78)	12 months (*n* = 66)
	*Mean (SE)*	*Mean (SE)*	*Mean (SE)*	*Mean (SE)*	*Mean (SE)*	*Mean (SE)*
GF:S	5.89 (0.16)	6.42 (0.16)^a^^**^	6.52 (0.19)^a^^**^	5.93 (0.14)	6.21 (0.15)	6.34 (0.17)
GF:R	5.97 (0.28)	6.62 (0.26)	6.19 (0.28)^b^^**^	5.27 (0.26)	5.80 (0.24)	6.07 (0.26)^a^^*^
DPAS	55.43 (2.32)	49.89 (2.21)^a^^*^	49.20 (2.45)	55.27 (2.16)	53.86 (2.07)	53.05 (2.28)
SOPS +	9.89 (0.50)	6.83 (0.50)^a^^***^	5.91 (0.51)^a^^***^	10.78 (0.46)	8.16 (0.46)^a^^***^	6.86 (0.47)^a^^***^^b^^**^
SOPS −	11.11 (0.76)	8.58 (0.74)^a^^**^	8.14 (0.80)^a^^***^	11.87 (0.70)	9.93 (0.68)^a^^*^	9.41 (0.74)^a^^**^
CDSS	6.16 (0.61)	4.15 (0.50)^a^^*^	3.35 (0.44)^a^^***^	6.02 (0.56)	4.30 (0.46)^a^^*^	3.25 (0.41)^a^^***^
SAS	40.49 (1.49)	36.70 (1.47)^a^^*^	35.60 (1.47)^a^^**^	40.28 (1.39)	36.75 (1.37)^a^^*^	36.17 (1.36)^a^^*^
SIAS	35.37 (2.34)	30.09 (2.35)^a^^*^	28.71 (2.23)^a^^**^	35.23 (2.18)	32.48 (2.20)	32.43 (2.06)
SSES	53.04 (2.81)	59.27 (2.86)^a^^*^	57.85 (2.93)	51.71 (2.59)	56.56 (2.69)	56.03 (2.71)
ABS	7.35 (0.41)	6.94 (0.42)	6.92 (0.45)	7.52 (0.38)	7.05 (0.39)	7.25 (0.41)
BCSS other	7.87 (0.74)	6.93 (0.72)	5.27 (0.76)^a^^*^	6.72 (0.69)	6.21 (0.68)	7.00 (0.69)
BCSS self	6.62 (0.80)	5.91 (0.79)	4.36 (0.70)	7.88 (0.74)	6.68 (0.74)	5.73 (0.64)^a^^*^

*Note:* Mean represents the least squares means estimated by the generalized linear model, SE represents the standard error of the mean; GF:S, Global Functioning: Social; GF:R, Global Functioning: Role; SOPS +, Scale of Psychosis-Risk Symptoms Positive symptoms; SOPS -, Scale of Psychosis-Risk Symptoms Negative symptoms; CDSS, Calgary Depression Scale for Schizophrenia; SAS, Social Anxiety Scale; SIAS, Social Interaction Anxiety Scale; DPAS, Defeatist Performance Beliefs Scale; SSES, Social Self-Efficacy Scale; ABS, Asocial Beliefs Scale; BCSS, Brief Core Schema Scale, CBSST, Cognitive-Behavioral Social Skills Training; ST, supportive therapy.

Significance:

^a^significantly different from baseline;

^b^significantly different from end of treatment. *P ≤ .05, **P ≤ .01, ***P ≤ .001

There were some significant within-group changes over time. For the primary and secondary outcomes, both groups had some small improvements in role functioning and the CBSST group showed significant improvements in social functioning, and defeatist attitudes. However, the mean improvements in social and role functioning were less than one point on the GF: social and role scales. Both groups showed significant improvement on positive and negative symptoms, depression, and anxiety. In both groups, there were a few small improvements on the Brief Core Schema Scales and self-efficacy scales.

We repeated the mixed model analyses controlling first for number of sessions attended, for IQ and for medication. The results were the same. See supplementary tables 5–7. The interaction effects between (1) number of sessions and (2) IQ and the treatment groups were checked and there was no minimum number of sessions someone needed to attend and no minimum score of IQ someone needed to have for the treatment to be effective. We also conducted the analysis on the total sample of 203 for the primary and secondary outcomes and the results were similar. See supplementary table 8.

### Transition to Psychosis

During the study, 5 participants made the transition to psychosis. One participant did not attend group, one only attended 3 sessions, and 2 ST participants and one CBSST participant made the transition before the end of their respective group treatment. Four completed the 12-month assessment and only one had a symptom at the psychotic level at 12 months. A Kaplan–Meier analysis was conducted to report the time to transition and is presented in supplementary figures 1 and 2. Figure 1 depicts time to onset of psychosis survival probability from baseline to transition by groups. Mean days to transition for the CBSST group were 423 days. Mean days to transition for the ST group were 171.3 days, and the log-rank analyses were nonsignificant (χ2 =2.38, *P* = .1228). Supplementary figure 2 depicts time to onset of psychosis survival probability from baseline to transition for all participants. Mean days to transition were 223.6 days.

## Discussion

This article presents the results of an RCT comparing the effects of CBSST with a ST in a sample of CHR youth. The key outcome measures were social and role functioning, areas in which CHR youth are known to have difficulties. Defeatist performance attitudes were the secondary outcome. As exploratory analyses, we examined positive and negative symptoms, anxiety, and depression, plus measures of self-efficacy and beliefs about the self. At baseline, on average, both groups presented with moderate impairment in social and role functioning, low levels of negative symptoms, and self-reported depression and anxiety. Ratings on the self-perception measures reflected that the sample was presented with some difficulties on these measures relative to what is observed in healthy controls.

There were no significant between-group differences in any outcome measure. For the key outcomes, there were statistically significant within-group improvements for CBSST in social functioning and defeatist beliefs and for both groups in role functioning. However, the within-group changes in social and role functioning have to be viewed with caution as on average the changes were less than one point which suggests no clinical significance. Both groups showed significant improvement in positive and negative symptoms, depression, and anxiety. There was minimal if any change in the self-belief measures.

It is possible that both group therapy interventions resulted in improvement in clinical symptoms in that being with peers with similar symptomatic difficulties and sharing these difficulties may be helpful, and for these young people, the nonspecific therapeutic elements found in group work may have had an advantage. However, one can only speculate as there is no comparison or wait-list group with which to compare.

The strengths of this study are that it was a well-conducted trial. Protections against sources of bias were well-maintained. Treatment was manualized. Therapists were well-trained and received ongoing supervision and peer support. Fidelity to treatment was monitored with good results. Raters were well-trained, met ongoing reliability on measures administered, and were blind to the treatment allocation. Principal investigators met every 4 months for the duration of the trial with a NIMH-approved DSMB. It was also one of the first RCTs that aimed to address social and role functioning.

However, there are some limitations. First, although our primary outcome measure was designed for a CHR youth population, our secondary outcome measure, the Defeatist Performance Attitude Scale, was not. Our self-report measures have been used with adolescent and emerging adult populations and with CHR populations but have not necessarily been validated. This was one of the conclusions of a recent review of Patient Reported Outcome Measures used with CHR populations.^43^ Secondly, we did not collect any qualitative data related to tolerability or group satisfaction. However, the mean number of sessions attended was 13.8 out of a possible 18 for both groups. In individual CBT trials, the mean number of sessions attended is between 10 and 12 out of a possible 20 and 26.^44–46^

Thirdly, the study was most likely underpowered as we had planned 62 per arm at the final follow-up and we ended up with 57 and 62. Furthermore, despite original power calculations, more participants may have been needed to detect a difference. Next, it is possible that the groups may have worked better as 18-week closed groups versus having rolling starts every 6 weeks to fit with the CBSST modules. It was a compromise between completely open and completely closed groups to avoid making potential CHR participants who are hard to find wait 18 weeks before starting a new group. Finally, there were 3 CBSST modules and participants who dropped out prior to 18 weeks would have not necessarily participated in the same modules. One option for future work might be to limit the sessions to what might be the most relevant, for example, around 12 sessions for this population and thus have a shorter and closed group.

In summary, the key observation from this study is that regardless of the group therapy participants improved symptomatically but not in terms of social and role functioning. But without a control or wait-list group, these improvements cannot be attributed to the therapies. To date, although there is a large CHR literature, little focuses on treatment trials, especially RCTs.^4^ To the best of our knowledge there are less than 30 published RCTs with the minority using a psychological treatment. Individual CBT has shown to be effective for improving attenuated psychotic symptoms and reducing transition rates^47–49^ but there are no CBT group studies with which to compare. As discussed in the introduction, RCTs reporting on social functioning have either found no social functioning change or a small change in both treatments. In addition, social functioning was not the target outcome. The one exception was a small pilot RCT of integrated social-cognitive and cognitive remediation that demonstrated an improvement of 1.1 points on the GF: Social Scale at 3 months.

Despite the lack of significant results in this study, it is still important to aim to improve social functioning, one of the most observed difficulties in CHR and one of the most observed predictors of later transition to psychosis. There are several possibilities for future directions. CBSST may be a useful treatment but only using selected modules that may be more relevant for the CHR population and thus allow for a shorter closed group. All aspects of this study were conducted in-person pre-COVID. However, with the increased use of virtual assessments, this might allow for greater compliance with follow-up assessments. Social-cognitive remediation may also be a future avenue to pursue.

Interestingly, recent studies have provided support for heterogeneous trajectories in CHR outcomes. For example, in one study^50^ using latent profile analysis, 3 separate classes of at-risk individuals emerged. Class 1 had a transition rate of 5.6%, with low scores on attenuated psychotic symptoms, and depression and intact neurocognition. Class 2 were “paranoid-affective” and had high levels of suspiciousness, mild negative symptoms, moderate depression, and a 14.2% transition rate. Class 3 was described as “negative-neurocognitive” and had the highest levels of negative symptoms, as well as the greatest level of neurocognitive, social-cognitive, and functional impairment and a transition rate of 29.3%. The second,^20^ using group-based multi-trajectory modeling, described 3 distinct profiles that were observed in a large sample of CHR individuals. The first group evidenced rapid symptomatic and functional improvement with 50% having good outcomes in symptoms and functioning; the second group demonstrated moderate improvement across symptom and functioning domains with only 25% reaching favorable outcomes; and in contrast, the third group exhibited moderate to severe impairment in symptoms and functioning that persisted and did not reach any remission criteria. Although the profiles vary in these articles, they all describe 3 outcome trajectories mild, moderate, and severe. Thus, it may help to consider different outcome trajectories when selecting participants for different treatments, in this case intervening for impaired functioning.

In conclusion, although there was symptomatic improvement, on average very little improvement in social functioning was observed as a result of either of these group treatments. A newly published review^51^ proposes that impaired social functioning should be considered as a manifestation of schizophrenia and this may, in fact, also be the case for the at-risk for psychosis syndromes. This underscores further the importance of finding successful treatments for the impaired functioning so often observed in these young CHR individuals, particularly since its relevance as predictor of later transition to psychosis.

## Supplementary Material

sgad020_suppl_Supplementary_Material
