# Supplementary material for: Cognitive-Behavioral Social Skills Training: Outcome of a Randomized Controlled Trial for Youth at Risk of Psychosis
Source: Schizophr Bull Open. 2023 Aug 2;4(1):sgad020. doi: 10.1093/schizbullopen/sgad020 (PMC10439516; doi:10.1093/schizbullopen/sgad020)
Supplement: sgad020_suppl_Supplementary_Material [file sgad020_suppl_Supplementary_Material.docx]

**SUPPLEMENTARY MATERIAL**

**MANUAL ADAPTATION**

Cognitive-behavioral social skills training (CBSST) was adapted for younger individuals at clinical high-risk (CHR) for psychosis. In their CBSST manual for individuals with schizophrenia, Granholm et al. (2016) recommends that participants complete eighteen sessions and then possibly re-enroll for a second time, meaning they will attend thirty-six sessions in total. Because cognitive deficits are not as severe among the CHR population, a second exposure was not deemed necessary. Thus, the ReGroup participants completed the three 6-week modules at once. Most of the content was maintained from the original version, although some sessions covered the material in more detail or less detail. Changes included the types of activities and behavioral examples used, to make them more youth focused. Rapidly evolving youth culture meant facilitators tried to let youth contribute in ways that allowed examples and solutions discussed during group sessions to be current. For instance, while older populations might talk about calling a friend on the phone, most youth do not call people anymore. Instead, they might interact through text, or even limit communication to “snapping” on Snapchat. While some TV shows might be popular with adults, youth were more likely to talk about their favorite YouTuber or influencer. Facilitators strove to create a familiar and relaxed atmosphere in group sessions to prevent it from feeling like school. Finally, role and social functioning emerged organically and were themes often discussed in the groups denoting the importance of school, work, and social relationships for these youth.

Some examples of adaptations made in each module include but are not limited to:

**Goal setting:**

- The goal setting is covered in the first session of each module. The adapted manual guided participants to focus on living, learning, working and socializing areas of life (not symptoms) that tend to affect young people (e.g., school, family, work, romantic relationships, and friends). It is emphasized that goals should be valuable to the participant themself and not represent goals their parents might have for them. A further difference in the facilitation of goal setting sessions is that facilitators focused on developmentally relevant issues and events such as increasing autonomy from the family and strategizing steps related to career aspirations and adjusting to post-secondary education.

**Cognitive skills module:**

- Due to the cognitive deficits experienced by individuals with chronic schizophrenia, certain techniques included in the original manual were removed for the CHR population. For example, goal setting covered in the cognitive skills module (Session 1: *Introduction and goal setting*) was explained to the chronic population using the 7-7-7 Goal Jackpot mnemonic. This was removed for the CHR groups, where goal setting was only described as short- and long-term goals.
- The CHR manual includes a more detailed background of CBSST, unhelpful thoughts, and unusual experiences.
- Examples of “catching unhelpful thoughts” (Session 3: *The 3 C’s Catch-it, Check-it, Change-it*) were adapted from the original manual. While the original manual included some focus on symptom related experiences such as voices, paranoia, or being afraid of meeting someone new, the adapted manual focused more on learning new skills, thoughts regarding the school environment, going to a party, or being assertive.

● Most the cognitive skills were taught through games and exercises adapted to engage a younger population:

- “Jenga tower.” In this game, thoughts and feelings were written on wood blocks from the commercial Jenga game, and as they were pulled from the tower, the participant was asked to identify whether the block contained a thought or a feeling.
- “Catching mistakes in thinking.” In this game, a group member is tossed a crumpled piece of paper with an example thought for a specific mistake in thinking written on it (e.g., “I will never make a friend”) and are asked what mistake in thinking it is (e.g., fortune-telling).
- “Heads Up.” In this game, participants wore hats with a type of cognitive distortion (e.g., all or nothing thinking, fortune-telling, or emotional reasoning) written on it. Without knowing what their hat said, participants had to guess based on descriptive clues given to them by other group members.

**Social skills module:**

- Basic social skills were covered early in both versions of this module, although they were less pertinent for the CHR group. Considering this, some aspects were relaxed; for example, the instruction in the original manual to avoid staring directly at people was modified for the CHR population to advise them to maintain good eye contact.
- Many CHR participants had adequate basic social skills and could integrate instructions for appropriate communication into their role-play well. While the original manual stressed practicing role-play three times with feedback on each attempt, many CHR youth made the necessary changes by the second role-play. Facilitators would then increase the difficulty and use social skills role-play as an opportunity to practice addressing specific issues related to a participant’s goals. The participant could then apply the role-play experience to their “at-home practice” that week, as they worked towards achieving a goal. For example, if a participant were having an ongoing issue setting boundaries with a parent, they could role-play having a difficult conversation during a group with another group member standing in as the parent. Bolstered by that role-play, the participant could use the experience and skills from session 5 to attempt to initiate that conversation effectively and appropriately with their parents in real life.
- The original manual focused on effective communication, expression of pleasant and unpleasant feelings, making positive requests, and asking for help with their goals. While most of this was covered within the adapted manual, session content differed. The adapted manual did not include a session on how to ask for help with goals or on explicitly expressing positive emotions. Instead, it focused on skills such as starting, maintaining, and ending conversations, identifying, coping with, and communicating about a range of feelings, setting healthy boundaries, and identifying members of the individual’s support structure.

**Problem-solving module:**

- CHR participants are asked to engage more thoroughly with their problem-solving strategies. For example, after brainstorming a list of possible solutions, the group discussed whether solutions are “passive” or “active”, and the benefits and drawbacks of these divergent strategies. This session (*Session 2: Solving problems effectively*) was not covered in the original manual.
- Example of problems outlined in the manuals differ in the following ways:
- Medication use (remembering to take it, making decisions about what/when to take it) has a greater focus in the original manual than in the adapted version, as it is much less common in the CHR population to be taking psychotropic medication regularly.
- There is an increased focus on concerns related to socializing and peer/intimate relationships for the CHR population.
- Schoolwork and finding a first job (i.e., how to write a resume) are raised as possible issues for the CHR population.
- No mention of issues related to positive symptoms appears in the adapted CHR manual, whereas the original uses some examples (i.e., focus on perceptual abnormalities as a problem to address).
- There is an increased focus on difficulties achieving independence from parents among the CHR population.
- More time spent on more intricate problems among CHR youth.

**References**

Granholm, E., McQuaid, J., & Holden, J. (2016). *Cognitive Behavioral Social Skills Training for schizophrenia: A practical treatment guide*. Guilford Press.

**POWER ANALYSES**

Sample size for was computed using the program *Repeated Measures with Attrition:Sample Sizes for 2 groups* (RMASS2) based on the work of Hedeker and colleagues.^1^ Current recommendations ^2-4^ are that clinical trial sample size calculations be based on the clinical meaningfulness of the effect tested rather than on data from pilot studies. Pilot data discussed above indicates a one-point change for about 20% of CHR individuals with TAU. For the current study we consider an additional 1-unit change in either the GF:S or GF:R scores for the CBSST treatment group to be a clinically meaningful difference. For example, a change from a rating of 6 to 7 would mean a change from having moderate social impairment with few friends and difficulty in developing relationships to having normal relationships and meaningful relationships with only mild problems. Significance level was set to 0.025 (0.05/2) after Bonferonni correction accounting for the two primary outcomes (social and role functioning). To detect this difference with 80% power, a sample size of 62 completers is needed per group, assuming a standard deviation of 1.8 for the GF social functioning outcome at all-time points and an autoregressive covariance structure with correlation between sequential assessments set at 0.45. Standard deviation and correlation estimates used in the above calculation were more conservative than the estimates obtained from the 216 patients participating in the NAPLS1 study above and a separate sample of 173 patients enrolled in the Recognition and Prevention program at the Hillside site.^5^ Assuming a linear trend for the primary outcomes, the *contrast effect size* associated with the above calculation is 0.55. The contrast (c_j_ (j-2.5), j = 1,2,3,4) that we chose for this calculation provides a test of treatment by linear time interaction, or equivalently, the difference in the slopes of the mean responses between the two treatment groups.^6^ Granholm and colleagues^7^ reported interaction effect sizes of approximately 0.50, for the mean Independent Living Skills Survey (ILSS) scores. Note that the effect size that we used for our calculation is larger than that reported in Granholm et al since the population in their study consisted of individuals with chronic schizophrenia, a far more debilitated population than the CHR subjects under study here. To be conservative, we assume an approximately 40-45% dropout rate across the course of the study. We will therefore recruit 112 per group i.e., 75 per site will give us 225 overall. We believe that the effect sizes for the role functioning outcome will be equal or greater than social functioning outcome in our study. Hence the sample size based on the social outcome will be sufficient to detect differences in slopes for the role outcome as well.

1. Hedeker D, Gibbons RD, Waternaux C. Sample size estimations for repeated measures analysis in randomized clinical trials with missing data. *Journal of Educational and Behavioral Statistics* 1999;24:70-93.
2. Kraemer HC, Kupfer DJ. Size of treatment effects and their importance to clinical research and practice. *Biol Psychiatry* 2006;59:990-996.
3. Leon AC. Implications of clinical trial design on sample size requirements. *Schizophr Bull* 2008;34:664-669.
4. Thabane L, Ma J, Chu R et al. A tutorial on pilot studies: the what, why and how. *BMC Med Res Methodol* 2010;10:1.
5. Cornblatt BA, Carrion RE, Addington J et al. Risk Factors for Psychosis: Impaired Social and Role Functioning. *Schizophr Bull* 2011. PMCID:PMC3494064
6. Lu K, Luo X, Chen PY. Sample size estimation for repeated measures analysis in randomized clinical trials with missing data*. Int J Biostat* 2008;4:Article.
7. Granholm E, Holden J, Link PC, McQuaid JR, Jeste DV. Randomized Controlled Trial of Cognitive Behavioral Social Skills Training for Older Consumers With Schizophrenia: Defeatist Performance Attitudes and Functional Outcome. *Am J Geriatr Psychiatry* 2012.

**Global Functioning: Social and Role Scales**

***Anchors for Global Functioning: Social Scale (GF:Social)***

10. Superior functioning in a wide range of social and interpersonal activities.

9. Good functioning in all social areas, and interpersonally effective.

8. Some transient mild impairment in social functioning.

7. Some persistent mild difficulty in social functioning.

6. Moderate impairment in social functioning.

5. Serious impairment in social functioning.

4. Major impairment in social functioning.

3. Marginal ability to function socially or maintain interpersonal relationships.

2. Unable to function socially or to maintain any interpersonal relationships.

1. Extreme social isolation.

***Anchors for Global Functioning: Role Scale (GF:Role)***

10. Superior role functioning

9. Above average role functioning

8. Good role functioning.

7. Mild impairment in role functioning.

6. Moderate impairment in role functioning.

5. Serious impairment in role functioning.

4. Major impairment in role functioning.

3. Marginal ability to function.

2. Inability to function.

1. Extreme role dysfunction.

| **Supplementary Table 1. Comparisons between participants with follow-up and retained in study and those who were not included in final analyses** | | | | | |
| --- | --- | --- | --- | --- | --- |
| **Variable** | | **Removed**  **n = 51** | **Retained**  **n = 152** | **Test Statistic** |  |
|  | | *Mean (SD)* | *Mean (SD)* | *t* | *P value* |
| Age (years) | | 17.31 (3.62) | 17.42 (4.06) | -0.18 | 0.859 |
| Years of education | | 10.51 (2.48) | 10.38 (2.65) | 0.30 | 0.762 |
| GF:S | | 5.84 (1.19) | 5.91 (1.29) | -0.32 | 0.753 |
| GF:R | | 5.04 (2.12) | 5.59 (2.35) | -1.49 | 0.139 |
| SOPS positive | | 11.31 (3.35) | 10.37 (4.21) | 1.45 | 0.147 |
| SOPS negative | | 13.33 (5.88) | 11.52 (6.34) | 1.80 | 0.073 |
| IQ | | 99.40 (14.45) | 103.50 (13.70) | -1.73 | 0.085 |
|  | | *Frequency (%)* | *Frequency (%)* | χ*^2^* | *P value* |
| Sex | |  |  |  |  |
|  | Male | 30 (58.8) | 69 (45.4) | 2.76 | 0.097 |
|  | Female | 21 (41.2) | 83 (54.6) |  |  |
| Race | |  |  |  |  |
|  | Caucasian | 34 (66.7) | 92 (60.5) | 0.75 | 0.686 |
|  | Black | 4 (7.8) | 17 (11.2) |  |  |
|  | Other^a^ | 13 (25.5) | 43 (28.3) |  |  |
| Marital status | |  |  |  |  |
|  | Single/never married | 48 (96) | 146 (96.1) | 1.61 | 0.447 |
|  | Married/common law | 2 (4.0) | 3 (2.0) |  |  |
| Living arrangement | |  |  |  |  |
|  | Living with family | 46 (90.2) | 134 (88.2) | 4.18 | 0.382 |
|  | Living with spouse/partner | 2 (3.9) | 8 (5.3) |  |  |
|  | Living on own | 0 (0.0) | 3 (2.0) |  |  |
|  | Living with others^b^ | 2 (3.9) | 7 (4.6) |  |  |
| Education Completed | |  |  |  |  |
|  | Grade school | 35 (68.6) | 101 (66.5) | 2.1 | 0.717 |
|  | High school | 13 (25.5) | 41 (27.0) |  |  |
|  | College | 2 (3.9) | 9 (5.9) |  |  |
|  | Technical school | 1 (2.0) | 1 (0.7) |  |  |
| Current employment | |  |  |  |  |
|  | Working full time | 3 (5.9) | 6 (4.0) | 0.73 | 0.867 |
|  | Working part-time | 7 (13.7) | 24 (15.8) |  |  |
|  | Worked in past year | 9 (17.7) | 32 (21.1) |  |  |
|  | Not worked in past year | 32 (62.8) | 90 (59.2) |  |  |
| *^a^ Includes First Nations, East Asian, Southeast Asian, South Asian, West/Central Asian, and Middle Eastern, Native Hawaiian or Pacific Islander, Interracial*  *^b^ Includes living with friends (excluding spouse/partners), in a boarding/group home, or academic residence.*  *GF:S, Global Functioning: Social; GF:R, Global Functioning: Role; SOPS, Scale of Psychosis-Risk Symptoms.* | | | | | |

| **Supplementary Table 2. Comparisons between CBSST participants with follow-up and retained in study and those who were not included in final analyses** | | | | | |
| --- | --- | --- | --- | --- | --- |
| **Variable** | | **Removed**  **n = 29** | **Retained**  **n = 70** | **Test Statistic** |  |
|  | | *Mean (SD)* | *Mean (SD)* | *t* | *P value* |
| Age (years) | | 17.03 (3.72) | 17.36 (4.01) | -0.37 | 0.711 |
| Years of education | | 10.13 (2.28) | 10.30 (2.68) | -0.29 | 0.776 |
| GF:S | | 5.79 (1.15) | 5.89 (1.34) | -0.33 | 0.745 |
| GF:R | | 5.37 (1.93) | 5.97 (2.21) | -1.26 | 0.212 |
| SOPS positive | | 11.62 (3.27) | 9.89 (4.60) | 2.12 | 0.038 |
| SOPS negative | | 13.14 (5.66) | 11.11 (5.42) | 1.67 | 0.098 |
| IQ | | 99.20 (11.37) | 103.00 (12.24) | -1.35 | 0.181 |
|  | | *Frequency (%)* | *Frequency (%)* | χ*^2^* | *P value* |
| Sex | |  |  |  |  |
|  | Male | 19 (65.52) | 29 (41.43) | 4.76 | 0.029 |
|  | Female | 10 (34.48) | 41 (58.57) |  |  |
| Race | |  |  |  |  |
|  | Caucasian | 20 (68.97) | 41 (58.57) | 1.03 | 0.599 |
|  | Black | 2 (6.90) | 8 (11.43) |  |  |
|  | Other^a^ | 7 (24.14) | 21 (30.00) |  |  |
| Marital status | |  |  |  |  |
|  | Single/never married | 28 (100.00) | 67 (95.71) | 1.24 | 0.539 |
|  | Married/common law | 0 (0.00) | 3 (4.29) |  |  |
| Living arrangement | |  |  |  |  |
|  | Living with family | 27 (93.1) | 63 (90.00) | 2.92 | 0.404 |
|  | Living with spouse/partner | 0 (0.00) | 3 (4.29) |  |  |
|  | Living on own | 0 (0.00) | 2 (2.86) |  |  |
|  | Living with others^b^ | 2 (6.90) | 2 (2.86) |  |  |
| Education Completed | |  |  |  |  |
|  | Grade school | 22 (75.86) | 48 (68.57) | 4.15 | 0.386 |
|  | High school | 6 (20.69) | 19 (27.14) |  |  |
|  | College | 0 (0.00) | 2 (2.86) |  |  |
|  | Technical school | 1 (3.45) | 1 (1.43) |  |  |
| Current employment | |  |  |  |  |
|  | Working full time | 1 (3.45) | 2 (2.86) | 0.27 | 0.965 |
|  | Working part-time | 5 (17.24) | 15 (21.43) |  |  |
|  | Worked in past year | 7 (24.14) | 15 (21.43) |  |  |
|  | Not worked in past year | 16 (55.17) | 38 (54.29) |  |  |
| *^a^ Includes First Nations, East Asian, Southeast Asian, South Asian, West/Central Asian, and Middle Eastern, Native Hawaiian or Pacific Islander, Interracial*  *^b^ Includes living with friends (excluding spouse/partners), in a boarding/group home, or academic residence.*  *GF:S, Global Functioning: Social; GF:R, Global Functioning: Role; SOPS, Scale of Psychosis-Risk Symptoms.* | | | | | |

| **Supplementary Table 3. Comparisons between ST participants with follow-up and retained in study and those who were not included in final analyses** | | | | | |
| --- | --- | --- | --- | --- | --- |
| **Variable** | | **Removed**  **n = 22** | **Retained**  **n = 82** | **Test Statistic** |  |
|  | | *Mean (SD)* | *Mean (SD)* | *t* | *P value* |
| Age (years) | | 17.68 (3.55) | 17.49 (4.11) | 0.20 | 0.841 |
| Years of education | | 11.00 (2.69) | 10.45 (2.64) | 0.86 | 0.390 |
| GF:S | | 5.91 (1.27) | 5.93 (1.26) | -0.06 | 0.954 |
| GF:R | | 4.59 (2.30) | 5.27 (2.44) | -1.17 | 0.245 |
| SOPS positive | | 10.91 (3.49) | 10.78 (3.83) | 0.14 | 0.887 |
| SOPS negative | | 13.59 (6.58) | 11.87 (7.04) | 1.04 | 0.300 |
| IQ | | 99.67 (18.25) | 104.00 (14.90) | -1.08 | 0.282 |
|  | | *Frequency (%)* | *Frequency (%)* | χ*^2^* | *P value* |
| Sex | |  |  |  |  |
|  | Male | 11 (50.00) | 40 (48.78) | 0.01 | 0.919 |
|  | Female | 11 (50.00) | 42 (51.22) |  |  |
| Race | |  |  |  |  |
|  | Caucasian | 14 (63.64) | 51 (62.20) | 0.07 | 0.968 |
|  | Black | 2 (9.09) | 9 (10.98) |  |  |
|  | Other^a^ | 6 (27.27) | 22 (26.83) |  |  |
| Marital status | |  |  |  |  |
|  | Single/never married | 20 (90.91) | 79 (96.34) | 4.32 | 0.116 |
|  | Married/common law | 2 (9.09) | 3 (3.66) |  |  |
| Living arrangement | |  |  |  |  |
|  | Living with family | 19 (86.36) | 71 (86.59) | 5.57 | 0.234 |
|  | Living with spouse/partner | 2 (9.09) | 5 (6.10) |  |  |
|  | Living on own | 0 (0.00) | 1 (1.22) |  |  |
|  | Living with others^b^ | 1 (4.55) | 5 (6.10) |  |  |
| Education Completed | |  |  |  |  |
|  | Grade school | 13 (59.06) | 53 (64.63) | 1.58 | 0.813 |
|  | High school | 7 (31.82) | 22 (26.83) |  |  |
|  | College | 0 (0.00) | 2 (2.44) |  |  |
|  | Technical school | 2 (9.09 | 5 (6.1) |  |  |
| Current employment | |  |  |  |  |
|  | Working full time | 2 (9.09) | 4 (4.88) | 2.10 | 0.550 |
|  | Working part-time | 2 (9.09) | 9 (10.98) |  |  |
|  | Worked in past year | 2 (9.09) | 17 (20.73) |  |  |
|  | Not worked in past year | 16 (72.73) | 52 (63.41) |  |  |
| *^a^ Includes First Nations, East Asian, Southeast Asian, South Asian, West/Central Asian, and Middle Eastern, Native Hawaiian or Pacific Islander, Interracial*  *^b^ Includes living with friends (excluding spouse/partners), in a boarding/group home, or academic residence.*  *GF:S, Global Functioning: Social; GF:R, Global Functioning: Role; SOPS, Scale of Psychosis-Risk Symptoms.* | | | | | |

| **Supplementary Table 4: Medications** | | | | | | | | | |
| --- | --- | --- | --- | --- | --- | --- | --- | --- | --- |
|  | ***Baseline*** | | | ***End of Treatment*** | | | ***12-months*** | | |
| ***Medication*** | ***Number***  ***taking*** | ***mean***  ***Dose*** | ***CPZ***  ***equivalent*** | ***Number***  ***taking*** | ***mean***  ***Dose*** | ***CPZ***  ***equivalent*** | ***Number***  ***taking*** | ***mean***  ***Dose*** | ***CPZ***  ***equivalent*** |
| Aripiprazole | 9 | 8.6mg | 115 mg | 7 | 13 mg | 173 mg | 7 | 11.7 mg | 167 mg |
| Risperidone | 11 | 1.5 mg | 150 mg | 9 | 2.5 mg | 250 mg | 9 | 2 mg | 200 mg |
| Quetiapine | 12 | 177 mg | 236 mg | 8 | 163 mg | 217 mg | 10 | 200 mg | 267 mg |
| Lurasidone | 2 | 60 mg | 375 mg | 4 | 32.5 mg | 203 mg | 2 | 60 mg | 375 mg |
| Olanzapine | 0 | 0 | 0 | 0 | 0 | 0 | 1 | 10 mg | 200 mg |
|  | | | | | | | | | |
| *Total number* | 34 |  |  | 28 |  |  | 29 |  |  |
| *Number in CBSST:ST* | 16:18 |  |  | 16:12 |  |  | 17:11 |  |  |

| **Supplementary Table 5. Differences in variables within and between groups controlling for number of sessions attended** | | | | | | |
| --- | --- | --- | --- | --- | --- | --- |
| Variables | CBSST (N=70) | | | ST (N=82) | | |
|  | Baseline  (n=70) | End of Treatment  (n=66) | 12 months  (n=57) | Baseline  (n=82) | End of Treatment  (n=78) | 12 months  (n=66) |
|  | *Mean (SE)* | *Mean (SE)* | *Mean (SE)* | *Mean (SE)* | *Mean (SE)* | *Mean (SE)* |
| GF:S | 5.89 (0.16) | 6.42 (0.16)^a**^ | 6.52 (0.19)^a**^ | 5.93 (0.14) | 6.20 (0.15) | 6.34 (0.17) |
| GF:R | 5.97 (0.28) | 6.62 (0.26) | 6.19 (0.28) | 5.27 (0.26) | 5.80 (0.24) | 6.07 (0.26)^a*^ |
|  |  |  |  |  |  |  |
| DPAS | 55.41 (2.33) | 49.88 (2.21)^a*^ | 49.17 (2.21) | 55.21 (2.17) | 53.83 (2.07) | 53.05 (2.27) |
|  | | | | | | |
| SOPS + | 9.88 (0.50) | 6.83 (0.50)^a***^ | 5.91 (0.51)^a***^ | 10.77 (0.46) | 8.15 (0.46)^a***^ | 6.85 (0.47)^a***b**^ |
| SOPS - | 11.11 (0.76) | 8.58 (0.74)^a**^ | 8.13 (0.81)^a***^ | 11.84 (0.70) | 9.91 (0.68)^a*^ | 9.40 (0.75)^a**^ |
| CDSS | 6.16 (0.61) | 4.15 (0.50)^a*^ | 3.36 (0.44)^a***^ | 6.03 (0.56) | 4.31 (0.46)^a*^ | 3.24 (0.41)^a***^ |
| SAS | 40.49 (1.49) | 36.70 (1.48)^a*^ | 35.60 (1.47)^a**^ | 40.29 (1.40) | 36.76 (1.37)^a*^ | 36.17 (1.36)^a*^ |
| SIAS | 35.33 (2.34) | 30.06 (2.34)^a*^ | 28.67 (2.23)^a**^ | 35.15 (2.18) | 32.42 (2.20) | 32.40 (2.06) |
|  |  |  |  |  |  |  |
| SSES | 53.04 (2.82) | 59.28 (2.87)^a*^ | 57.86 (2.93) | 51.73 (2.61) | 56.57 (2.69) | 56.04 (2.71) |
| ABS | 7.35 (0.41) | 6.94 (0.42) | 6.92 (0.45) | 7.51 (0.38) | 7.04 (0.40) | 7.25 (0.41) |
| BCSS other | 7.86 (0.74) | 6.93 (0.72) | 5.25 (0.75)^a*^ | 6.70 (0.69) | 6.20 (0.68) | 7.00 (0.69) |
| BCSS self | 6.62 (0.80) | 5.91 (0.79) | 4.37 (0.70) | 7.89 (0.74) | 6.68 (0.74) | 5.73 (0.64)^a*^ |
| Abbreviations: Mean represents the least squares means estimated by the generalized linear model, SE represents the standard error of the mean; GF:S, Global Functioning: Social; GF:R, Global Functioning: Role; SOPS +, Scale of Psychosis-Risk Symptoms Positive symptoms; SOPS -, Scale of Psychosis-Risk Symptoms Negative symptoms; CDSS, Calgary Depression Scale for Schizophrenia; SAS, Social Anxiety Scale; SIAS, Social Interaction Anxiety Scale; DPAS, Defeatist Performance Beliefs Scale; SSES, Social Self Efficacy Scale; ABS, Asocial Beliefs Scale; BCSS, Brief Core Schema Scale.  Significance: a= significantly different from baseline; b= significantly different from end of treatment. *p<0.05, **p<0.01, ***p<0.001 | | | | | | |

| **Supplementary Table 6. Differences in variables within and between groups controlling for IQ** | | | | | | | |
| --- | --- | --- | --- | --- | --- | --- | --- |
| Variables | | CBSST (N=70) | | | ST (N=82) | | |
|  | | Baseline  (n=70) | End of Treatment  (n=66) | 12 months  (n=57) | Baseline  (n=82) | End of Treatment  (n=78) | 12 months  (n=66) |
|  | | *Mean (SE)* | *Mean (SE)* | *Mean (SE)* | *Mean (SE)* | *Mean (SE)* | *Mean (SE)* |
| GF:S | | 5.88 (0.16) | 6.42 (0.16)^a**^ | 6.51 (0.19)^a**^ | 5.93 (0.15) | 6.19 (0.15) | 6.32 (0.17) |
| GF:R | | 5.98 (0.28) | 6.62 (0.26) | 6.19 (0.28) | 5.26 (0.26) | 5.77 (0.24) | 6.05 (0.26) |
|  | |  |  |  |  |  |  |
| DPAS | | 55.48 (2.31) | 49.94 (2.19)^a*^ | 49.25 (2.45) | 55.62 (2.17) | 54.26 (2.06) | 53.26 (2.29) |
|  |  |  |  |  |  |  |  |
| SOPS + | | 9.89 (0.50) | 6.84 (0.50)^a***^ | 5.92 (0.51)^a***^ | 10.84 (0.47) | 8.22 (0.46)^a***^ | 6.94 (0.47)^a***b*^ |
| SOPS - | | 11.11 (0.76) | 8.58 (0.74)^a**^ | 8.14 (0.80)^a***^ | 11.99 (0.70) | 10.04 (0.69)^a*^ | 9.53 (0.75)^a**^ |
| CDSS | | 6.17 (0.60) | 4.16 (0.49)^a*^ | 3.37 (0.44)^a***^ | 6.08 (0.56) | 4.34 (0.46)^a*^ | 3.28 (0.42)^a***^ |
| SAS | | 40.46 (1.48) | 36.68 (1.48)^a*^ | 25.58 (1.48)^a**^ | 40.51 (1.40) | 36.92 (1.38)^a*^ | 36.32 (1.38)^a*^ |
| SIAS | | 35.37 (2.34) | 30.09 (2.36)^a*^ | 28.70 (2.23)^a**^ | 25.56 (2.19) | 32.66 (2.22) | 32.74 (2.07) |
|  | |  |  |  |  |  |  |
| SSES | | 53.01 (2.81) | 59.14 (2.87)^a*^ | 57.81 (2.93) | 51.38 (2.61) | 56.25 (2.71) | 55.69 (2.73) |
| ABS | | 7.35 (0.41) | 6.94 (0.42) | 6.92 (0.45) | 7.59 (0.38) | 7.12 (0.40) | 7.33 (0.42) |
| BCSS other | | 7.83 (0.72) | 6.90 (0.72) | 5.22 (0.75)^a*^ | 6.84 (0.68) | 6.30 (0.68) | 7.06 (0.70) |
| BCSS self | | 6.64 (0.81) | 5.93 (0.78) | 4.39 (0.69) | 7.94 (0.75) | 6.74 (0.74) | 5.78 (0.64)^a*^ |
| Abbreviations: Mean represents the least squares means estimated by the generalized linear model, SE represents the standard error of the mean; GF:S, Global Functioning: Social; GF:R, Global Functioning: Role; SOPS +, Scale of Psychosis-Risk Symptoms Positive symptoms; SOPS -, Scale of Psychosis-Risk Symptoms Negative symptoms; CDSS, Calgary Depression Scale for Schizophrenia; SAS, Social Anxiety Scale; SIAS, Social Interaction Anxiety Scale; DPAS, Defeatist Performance Beliefs Scale; SSES, Social Self Efficacy Scale; ABS, Asocial Beliefs Scale; BCSS, Brief Core Schema Scale.  Significance: a= significantly different from baseline; b= significantly different from end of treatment. *p<0.05, **p<0.01, ***p<0.001 | | | | | | | |

| **Supplementary Table 7. Differences in variables within and between groups controlling for medication** | | | | | | |
| --- | --- | --- | --- | --- | --- | --- |
| Variables | CBSST (N=70) | | | ST (N=82) | | |
|  | Baseline  (n=70) | End of Treatment  (n=66) | 12 months  (n=57) | Baseline  (n=82) | End of Treatment  (n=78) | 12 months  (n=66) |
|  | *Mean (SE)* | *Mean (SE)* | *Mean (SE)* | *Mean (SE)* | *Mean (SE)* | *Mean (SE)* |
| GF:S | 5.45 (0.29) | 6.42 (0.29)^a**^ | 6.49 (0.30)^a**^ | 5.38 (0.27) | 5.38 (0.27) | 6.50 (0.33)^a*^ |
| GF:R | 5.18 (0.54) | 6.61 (0.55) | 5.69 (0.47) | 4.39 (0.51) | 4.50 (0.61) | 5.92 (0.57) |
|  | | | | | | |
| DPAS | 56.93 (4.26) | 52.11 (4.18) | 48.83 (4.68) | 48.83 (4.17) | 48.61 (4.58) | 49.95 (5.00) |
|  | | | | | | |
| SOPS + | 11.34 (0.95) | 7.04 (1.07)^a**^ | 5.93 (0.95)^a***^ | 12.45 (0.90) | 7.89 (1.16)^a**^ | 8.61 (1.08)^a*^ |
| Abbreviations: Mean represents the least squares means estimated by the generalized linear model, SE represents the standard error of the mean; GF:S, Global Functioning: Social; GF:R, Global Functioning: Role; DPAS, Defeatist Performance Beliefs Scale; SOPS +, Scale of Psychosis-Risk Symptoms Positive symptoms.  Significance: a= significantly different from baseline; b= significantly different from end of treatment. *p<0.05, **p<0.01, ***p<0.001. | | | | | | |

| **Supplementary Table 8. Differences in clinical variables within and between groups using the total sample of 203.** | | | | | | |
| --- | --- | --- | --- | --- | --- | --- |
| Variables | CBSST (N=99) | | | ST (N=104) | | |
|  | Baseline  (n=99) | End of Treatment  (n=68) | 12 months  (n=58) | Baseline  (n=104) | End of Treatment  (n=80) | 12 months  (n=67) |
|  | *Mean (SE)* | *Mean (SE)* | *Mean (SE)* | *Mean (SE)* | *Mean (SE)* | *Mean (SE)* |
| GF:S | 5.86 (0.13) | 6.38 (0.15)^a**^ | 6.51 (0.17)^a***^ | 5.92 (0.12) | 6.21 (0.14) | 6.34 (0.16)^a*^ |
| GF:R | 5.80 (0.23) | 6.48 (0.24)^a*^ | 6.11 (0.26) | 5.13 (0.22) | 5.68 (0.23) | 5.99 (0.25) ^a*^ |
|  | | | | | | |
| DPAS | 55.55 (1.94) | 50.32 (2.07)^a*^ | 49.66 (2.36) | 54.45 (1.92) | 53.05 (1.99) | 52.24 (2.23) |
| Abbreviations: Mean represents the least squares means estimated by the generalized linear model, SE represents the standard error of the mean; GF:S, Global Functioning: Social; GF:R, Global Functioning: Role; DPAS, Defeatist Performance Beliefs Scale.  Significance: a= significantly different from baseline; b= significantly different from end of treatment. *p≤0.05, **p≤0.01. | | | | | | |


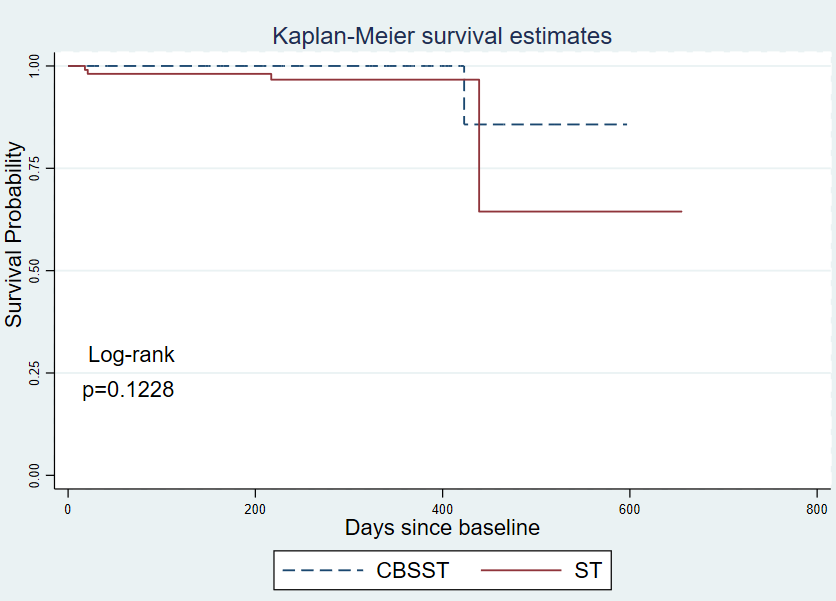


Supplementary Fig. 1. Kaplan–Meier survival estimates for CBSST and ST groups.


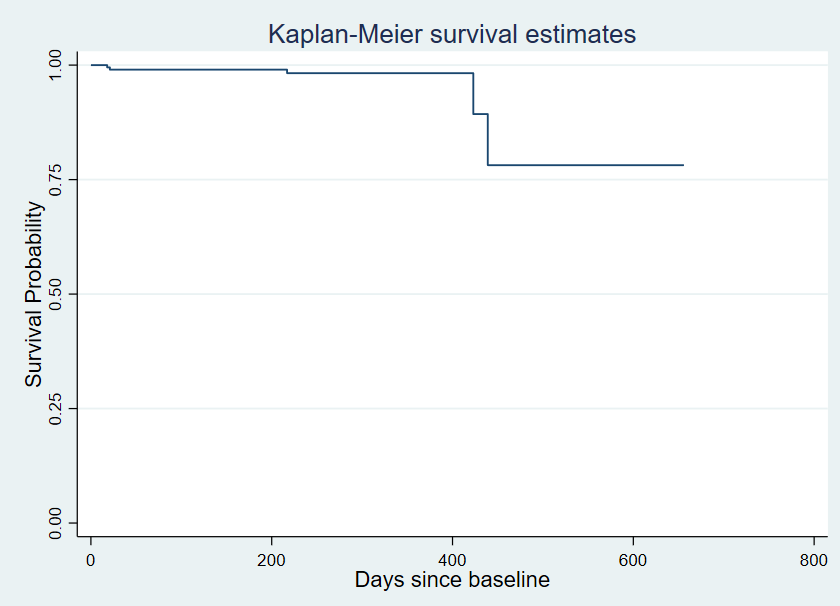


Supplementary Fig. 2. Kaplan–Meier survival estimates for all participants.
